# Micro-Cantilever Displacement Detection Based in Optical Fiber Tip

**DOI:** 10.3390/s19224826

**Published:** 2019-11-06

**Authors:** Paulo Robalinho, Orlando Frazão

**Affiliations:** INESC TEC and Department of Physics and Astronomy, Faculty of the University of Porto, Rua do Campo Alegre, 687 4169-007 Porto, Portugal

**Keywords:** physic, optic, sensor, detection, vibration, cantilever, microsphere, tip, optical fiber structure

## Abstract

This work demonstrates the potential of combining a microsphere with a tip for the functionality of the contact sensor. This sensor consists of a tip aligned with the fiber core and a microsphere, which appears during tip formation. This new structure was produced using the electric arc machine. The sensor operation consists of the variation of the tip curvature, which causes a variation of the optical paths and, consequently, a change in the output signal. The study of this micro-cantilever consisted of an exploration of the contact mode. In addition, the sensor was characterized by temperature, which shows very low sensitivity and vibration. This last characterization was performed with two configurations parallel and perpendicular to the oscillating surface. The perpendicular case showed higher sensitivity and has an operating band of 0 Hz to 20 kHz. In this configuration, for frequencies up to 2 Hz, the intensity varies linearly with the frequencies and with a sensitivity of 0.032 ± 0.001 (Hz^−1^). For the parallel case, the operating band was from 1.5 kHz to 7 kHz.

## 1. Introduction

At present, one of the most important techniques for the characterization of micro- and nanostructures is atomic force microcopy [1]. This consists of the existence of a tip, called the cantilever, that runs through a surface. A mirror is coupled to the cantilever which, using a laser beam, allows us to ascertain the profile of the sample [2]. A negative characteristic associated with this technique is that the cost of cantilever manufacturing is expensive, and cantilevers are weak. They also do not allow accurate information on the interaction mechanisms, due to the low speed of image acquisition [3], or on soft structures. 

Since the late twentieth century, some hypotheses have been studied to replace the metallic cantilever with an optical fiber [4]. In the literature, few fiber structures were studied, namely: The coupling of an optical fiber to existing metal cantilevers [5,6] and the potentialities of conical geometries in reflection mode (where results were not as accurate as those obtained by the metallic cantilever) [7,8,9] or in contact mode using structures such as the cantilever, which are fused to a cleaved fiber [10] and whose manufacturing processes are complex and have waveguides with misaligned sections whose manufacture is also complex [11,12]. In addition, the geometry presented in this paper reveals sensitivity toward vibrations. Most of the existing sensors can only measure frequencies up to 4 kHz [13,14,15]. However, there are already some sensors based on the variation phase that allow measuring frequencies up to 40 kHz [16].

This paper aims to present a geometry at the extremity of a fiber with the function of characterizing a given surface based on the intensity variation of the optical signal. This geometry was composed of a microsphere that resulted from the formation of the tip. This microsphere did not produce a variation in the optical signal because the dispersion was reduced and the junction between tip and microsphere was 38 µm. This new structure was characterized by contact with the perturbations of a surface and the temperature response, and, finally, the sensor was characterized in vibration. 

## 2. Materials and Methods

The sensor was produced using a conventional splice machine (“Sumitomo Electric™ Type-72C”, Osaka, Japan) in standard single mode fiber (SMF28). The interrogation systems used to characterize the geometry were an optical spectrum analyzer (OSA) (“Advantest Q8384”) with a resolution of 0.02 nm, a photodiode (‘Thorlabs PDA 10CS-EC’) with a 40 dB amplification, and an oscilloscope (‘Tektronix TDS 1002C-EDU’). The broad-spectrum source is centered at 1570 nm with a bandwidth of 100 nm.

**Figure 1:**
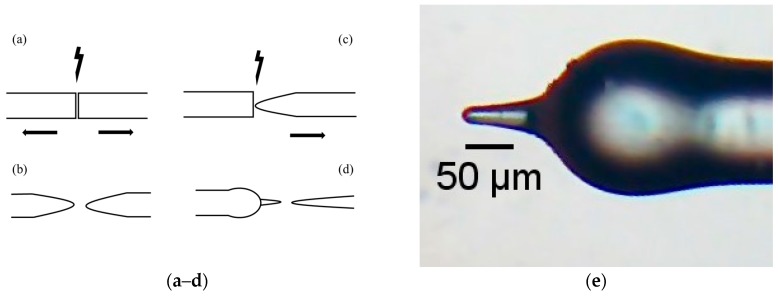
Manufacture steps (**a**–**d**) and head sensor (**e**).

The optical fiber micro-cantilever was produced by the following step in Figure 1a, in which the fusion of two cleaved fibers (with a duration of 10 s and a power of −40 a.u.) is presented and simultaneously applied a tension in the fibers; the final results are two fiber tips (Figure 1b), for the sensor only needs one fiber tip. In the Figure 1c, the fusion between a cleaved fiber and the fiber tip (with a duration of 10 s and a power of −40 a.u.) is shown. During the fusion, the fiber tip was under tension. The result was a narrow fiber tip (Figure 1d). In addition to the formation of the narrow fiber tip, a microsphere was formed and did not influence the performance of the sensor.

The geometry of the optical fiber micro-cantilever is shown in Figure 1e and consists of the microsphere with a diameter of 165 µm, a tip with a length of 82 µm, and a width of 38 µm in the widest zone and 13 µm in the thinnest zone. 

The main mechanical feature that allows the sensor to be a micro-cantilever is the flexibility of the tip, which is superior to the flexibility of the standard fiber because it has a much smaller diameter. Thus, the thinner the tip, the more mechanically sensitive it is. In addition, the higher the length of the tip, the higher the mechanical sensitivity. 

### 2.1. Contact Experimental Setup

**Figure 2:**
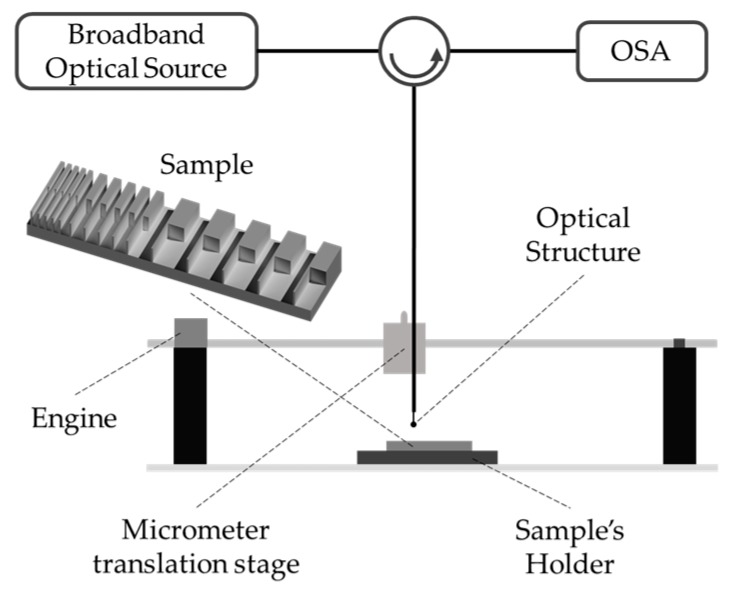
Assembly diagram for contact variation.

The sensitivity of the sensor in the detection of a surface profile in the contact mode is characterized. For this proposal, the setup of Figure 2 was used, and the grid features are shown in Table 1. The sensor was fixed by the optical fiber. The characterization began with the moving down of the optical fiber until the fiber tip contacted the grid elevations, causing a variation in the optical signal intensity. The fiber was moved to one of the grid ends. Finally, by means of the motor, with an average minimum speed of 3.75 mm/s, the fiber was moved along the grid. Thus, the curvatures to which the fiber tip will be submitted will not deform the fiber tip.

### 2.2. Temperature Setup

**Figure 3:**
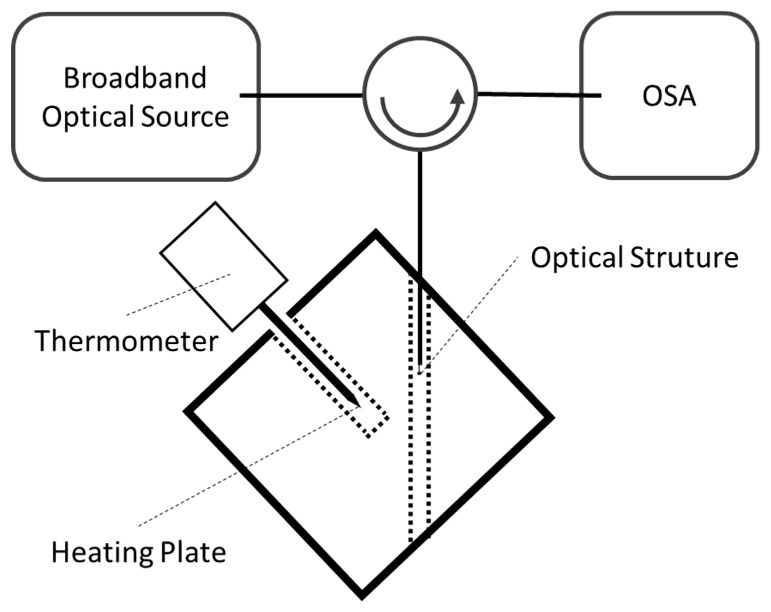
Assembly diagram for temperature variation.

In addition, the sensor was characterized in temperature and the schematic of Figure 3 was used. In this case, the optical fiber was placed inside a capillary tube where the optical fiber was the first to enter, following the geometry that was inside the capillary during the experimental execution. This procedure allowed safeguarding the physical integrity of the sensor during the entry and exit of the sensor from the heating plate.

### 2.3. Vibration Setup

**Figure 4:**
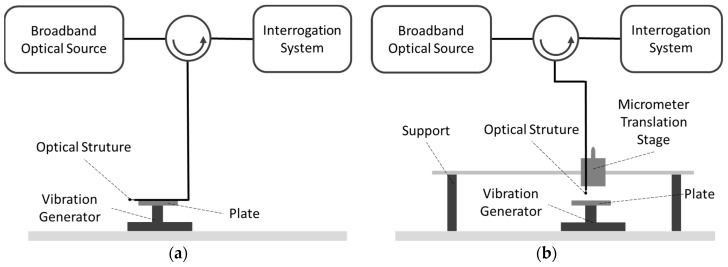
Assembly diagram for vibration with the sensor positioned: (**a**) Parallel and (**b**) perpendicular.

The optical fiber micro-cantilever sensor was studied when it was subjected to vibration. Figure 4 presents two setup configurations for two different sensor positions (parallel and perpendicular) in relation to oscillating plate. This oscillating plate was dark and was coupled with a vibration generator. As shown inn Figure 4a, the sensor was fixed to the plate by the optical fiber. The sensor was located outside the plate at 27.5 mm. As shown in Figure 4b, the sensor was fixed by the optical fiber in the micrometer translation stage and the contact with the oscillating surface was made by the fiber tip.

### 2.4. Optical Model

**Figure 5:**
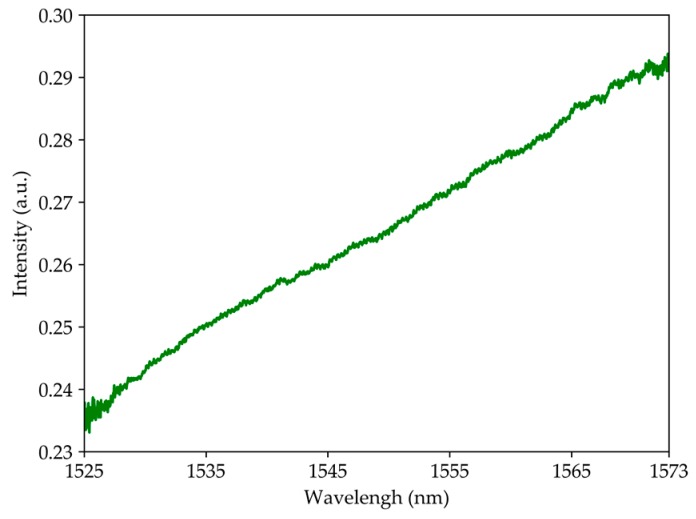
Output signal spectrum when geometry is suspended in air.

Figure 5 shows the spectrum of the output signal. As can be seen, the intensity increased with the wavelength close to the linear relationship, which indicates a possibility that the fringe has a dimension higher than 200 nm. Due to the limitation of the broadband source, it is not possible to observe the pattern fringe. However, it is very interesting to read this geometry as an intensity sensor. During the characterization, the microsphere did not influence the performance of the system, since the optical paths were mostly influenced by the fiber optic tip.

**Figure 6:**
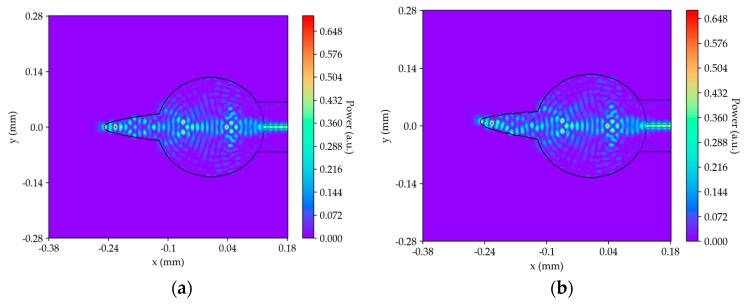
Frame 177 from two simulations: (**a**) Straight fiber tip and (**b**) curved fiber tip.

To complement the previous study, a simulation of the sensor performance was realized. Figure 6 shows two frames of the sensor performance simulation. For this, the “symmetric split step Fourier” method was used, and since there were no non-linear effects, the Hamiltonian is given by:

(1) $$H\;=\;\frac{1}{2}p^{2}+V$$

As shown in Figure 6a, the fiber tip was straight, which resulted in the formation of a symmetrical mode in relation to the fiber tip axis. As observed in Figure 6b, there was a change in the modes inside the curved fiber tip and the microsphere modes remained constant. Therefore, the performance of the sensor depended mainly on the fiber tip and the microsphere did not influence the sensor’s performance.

## 3. Results

In the characterization of the optical fiber micro-cantilever sensor, the contact mode was explored, as well as the temperature influence on the optical output signal and the sensor response when subjected to vibrations.

### 3.1. Fiber Tip Curvature

**Figure 7:**
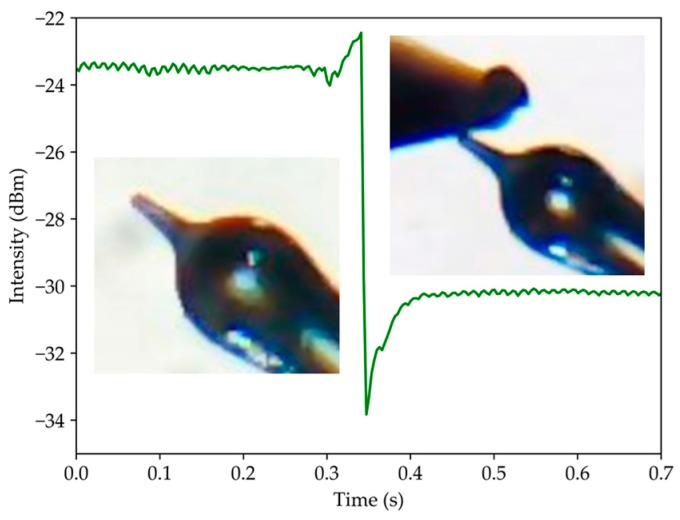
Sensor response when the tip is curved.

As described at the end of Section 2, the optical paths were mainly influenced by the tip. Figure 7 shows that when the tip was curved there was a variation in the optical output signal. In this case, the tip reached an angle of 2.5°, which resulted in a signal decrease of 7 dB. Thus, the sensor was very sensitive to the variation of the tip curvature. The decay time was 6 ms. This value corresponds to the response time of the system, to which the optical sensor was subjected. 

### 3.2. Contact Sensor

**Figure 8:**
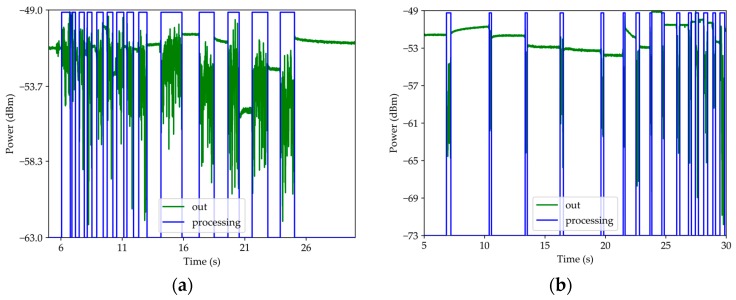
Sensor response for a grid scan: (**a**) path 1 and (**b**) path 2.

To determine this operating mode as a contact sensor, the setup in Figure 2 was used. Figure 8 shows a scan of each path of the grid: Figure 8a for path 1 and Figure 8b for path 2. The analysis focuses on the system response to wavelength of 1570 nm. Every time that the sensor contacted an elevation there was a signal change.

Figure 8 shows the optical signal, named “out”, and its processing by means of a computational algorithm, named “processing”. The algorithm consists in the analysis of the standard deviation, where each instant is considered as a “previous” or “next” instants. Thus, when the sensor is only surrounded by air, the signal is practically constant, and the standard deviation is minimal. When the sensor contacts a grid elevation or drags on an elevation, it implies that the curvature of the tip is in oscillation. Therefore, the optical signal contains this oscillation and the standard deviation is maximum. In addition, during sensor contact with grid perturbations, the average value of the optical signal is changed. This is due to the micrometric difference of the different elevation heights. For the 1 mm dimension, the standard deviation from the mean was 20%, for 2 mm was 9.1%, for 5 mm was 20%, and for 0.5 mm was 33% (Table 2). Soon this system revealed an ability to measure perturbation of the order of 2 mm. This standard deviation value is not associated with the sensitivity of the sensor but with the scan speed, which was not constant because the speed was at the lowest that the engine allowed and because the sensor was held by the optical fiber rather than the microsphere. Considering that the fastest acquisition time is 1 ms, the minimum spatial step is 7.5 µm.

### 3.3. Temperature Measurement

**Figure 9:**
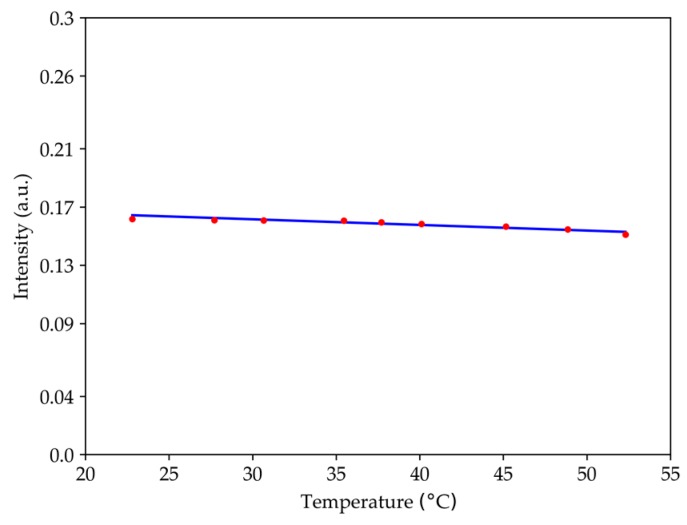
Optical signal intensity as a function of temperature for the wavelength of 1570 nm.

For this section, the assembly of the temperature setup of Figure 3 was used. The temperature range was from room temperature up to 55 °C. Figure 9 shows the response of the sensor at the wavelength of 1570 nm. The maximum variation is 0.01 which corresponds to 6.2% of the signal intensity at room temperature, and the sensibility was (3.9 ± 0.5) × 10^−4^/°C with a *r*^2^ of 0.90. Therefore, for the temperature range under study, the structure had very low sensitivity.

### 3.4. Vibration Sensor

**Figure 10:**
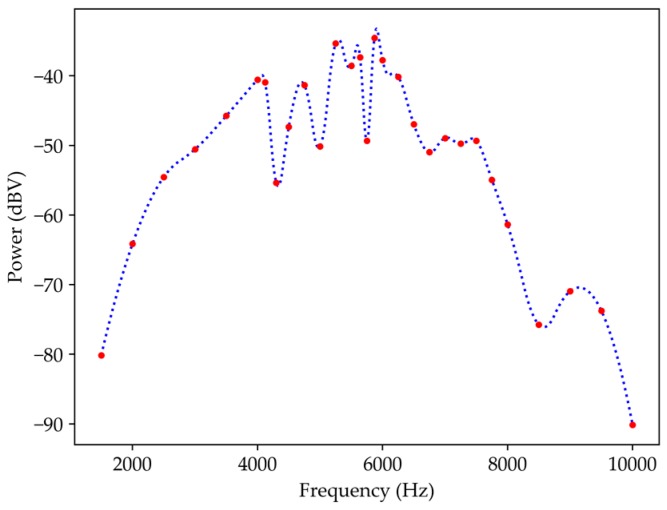
Transfer function in relation to an oscillating surface for a wavelength of 1570 nm and with the sensor parallel to the oscillating plate.

The response of this sensor when in contact with a periodical oscillating surface has also been studied. The amplitude of the mechanical oscillation of the surface was less than 0.5 mm. In this characterization, the sensor was placed in two positions, parallel and perpendicular, to the vibration generator. In the first case, the schema of Figure 4a was used, and resulted are shown in Figure 10, which presents the relationship between the electrical power and the frequency. For this purpose, the interrogation system was the photodetector and the intensity was converted into electrical power. The result of the sensor subject to vibration shows a similar response to a bandpass, i.e., for low frequencies below 1.5 kHz, the sensor was insensitive. In the flat region, the sensor shows several resonances resulting from the system used for the sensor characterization. Above 7 kHz there was a strong drop due to the limitation of the vibration generator.

**Figure 11:**
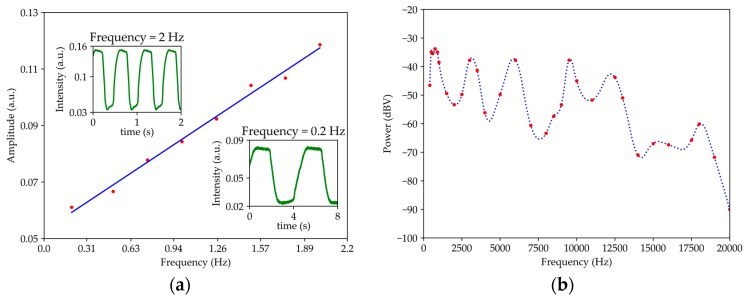
Transfer function in relation to an oscillating surface for a wavelength of 1570 nm and with the sensor perpendicular to the oscillating plate: (**a**) 0.2 Hz to 2 Hz and (**b**) 400 Hz to 20 kHz.

For the case of Figure 4b, where the sensor is perpendicular to the oscillating surface, Figure 11a,b shows the system response to low frequencies and the bandwidth between 400 Hz to 20 kHz. For frequencies between 0.2 Hz and 2 Hz (Figure 11a), the amplitude of the optical signal increases with increasing frequency, in addition to containing the mechanical oscillation frequency. However, the optical signal was saturated because the sensor was fixed by the optical fiber. When the tip arrives to the maximum curvature, the fiber starts to bend. The sensitivity for the frequency range under study is 0.032 ± 0.001 (Hz^−1^). For frequencies between 400 Hz and 20 kHz (see Figure 11b) present more sensitivity when compared to the parallel mode. However, higher resonances are presented with a similar maximum spectra power response due to the resonance of the tip.

## 4. Discussion and Conclusions

In summary, this sensor is sensitive to variations in the curvature of the tip, allowing us to ascertain the surface profile resulting in the contact mode. This mode allows us to evaluate the three dimensions. With the temperature characterization, the insensitive of sensor was proven. In addition, this optical fiber micro-cantilever allows determining the mechanical frequencies of a given system, and the case of the sensor parallel to the oscillating surface is less sensitive than the perpendicular case. Considering the measuring system used, for the parallel case the operating band is from 1.5 kHz to 7 kHz and for the perpendicular case the operating band is from 0 Hz to 20 kHz.

To increase the sensitivity of the geometry, the width of the tip should be reduced to be more flexible (i.e., higher mechanical sensitivity) and coated to increase the output signal power. However, reducing the size of the tip may lead to a single mode, which would imply less variation in the optical signal. To work around this problem, a multimode tip fused in series with a single mode tip should be used.

Thus, this geometrycan replace the current metallic cantilever. In addition, it is a good tool for micro-process characterization and is a good vibration sensor for micro-vibration systems, such as earthquake microwaves.

## Figures and Tables

**Table 1:** Grid feature: Square profile.

Period		Path 1		Path 2	
#	Length (mm)	Height (mm)	Length (mm)	Height (mm)	Length (mm)
5	10	5	5	0.5	5
5	4	2	5	0.5	5
5	2	1	5	0.5	5

**Table 2:** Data resulting from Figure 8.

Figure	Real Size	Measurement	
	(mm)	Average (mm)	SD (mm)
a	1	1.2	0.3
a	2	2.2	0.2
a	5	5	1
B	0.5	0.6	0.2
